# Adrenal Hemangioma: A Case of Retroperitoneal Tumor

**DOI:** 10.1155/2018/8796327

**Published:** 2018-02-19

**Authors:** Genta Iwamoto, Kota Shimokihara, Takashi Kawahara, Daiji Takamoto, Masahiro Yao, Jun-ichi Teranishi, Masako Otani, Hiroji Uemura

**Affiliations:** ^1^Department of Urology and Renal Transplantation, Yokohama City University Medical Center, Yokohama, Japan; ^2^Department of Urology, Yokohama City University Graduate School of Medicine, Yokohama, Japan; ^3^Division of Diagnostic Pathology, Yokohama City University Medical Center, Yokohama, Japan

## Abstract

**Introduction:**

Adrenal hemangioma is a rare disease, with only some 60 cases reported previously. Due to the difficulty of the preoperative diagnosis of adrenal hemangioma, almost all of the cases were diagnosed by a histopathological analysis of surgical specimens.

**Case Presentation:**

A 52-year-old man was referred to our department for further examination of his left retroperitoneal tumor. He had received hemodialysis due to chronic renal failure resulting from membranous nephropathy. Computed tomography revealed a mass around his left hilum. Magnetic resonance imaging (MRI) and positron-emission tomography (PET)-CT were unable to confirm or deny malignancy, and tumor markers, including CEA and CA19-9, showed slight elevation. His tumor grew from 38 mm to 54 mm in diameter in 7 months of follow-up. We therefore planned retroperitoneal tumor resection with left nephrectomy. Histopathologically, hyperplastic small vessels with hemorrhaging and denaturation were seen. The endothelial cells showed no variants or division of the nucleus. Based on this diagnosis, no further therapy was performed. He has had no recurrence in the eight months since the surgery.

**Conclusion:**

We herein report a rare case of adrenal hemangioma.

## 1. Introduction

Adrenal cystic disease is a rare entity, accounting for 2.3% of all adrenal tumors, and most such patients show no symptoms and are instead incidentally detected by computed tomography (CT) [1–3]. Foster et al. defined the following 4 groups of adrenal cystic disease: 45% with endothelial cyst (42% with lymphangioma and 3% with hemangioma), 39% with pseudocyst, 9% with epithelial cyst, and 7% with parasite cyst [1]. Adrenal hemangioma is a particularly rare disease. We herein report a case of retroperitoneal hemangioma incidentally detected by CT.

## 2. Case Presentation

A 52-year-old Asian man was referred to our department for further examination of his retroperitoneal tumor in April 2016. He had received hemodialysis due to grade 5 chronic kidney disease resulting from membranous nephropathy. His previous physician performed CT because the patient had lost 20 kg over the past 3 years, and CT revealed a left retroperitoneal mass. He had no remarkable physical findings. Laboratory data showed almost normal values, except for an elevated serum creatine level (4.31 mg/dL). A serum hormonal examination showed slight elevation in aldosterone as follows: cortisol 6.2 *μ*g/dL, aldosterone 410 pg/mL, adrenaline 19 pg/mL, noradrenaline 53 pg/mL, and dopamine 12 pg/mL. Tumor markers showed slight elevation of CEA, SCC, NSE, and IL-2 receptor as follows: CEA 5.7 ng/mL, AFP 7 ng/mL, CA125 27 U/mL, SCC 3.7 ng/mL, NSE 23.4 ng/mL, and IL-2 receptor 663.1 U/mL.

**Figure 1:**
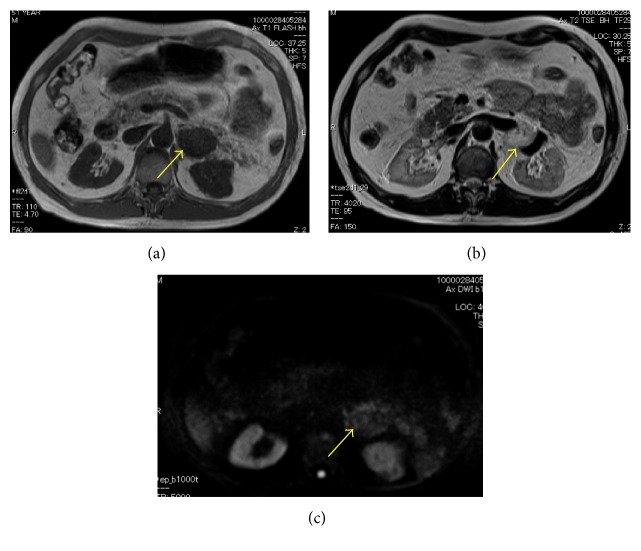
MRI images. (a) T1WI, (b) T2WI, and (c) DWI. The tumor (arrow) showed low intensity in T1WI and high intensity in T2WI.

**Figure 2:**
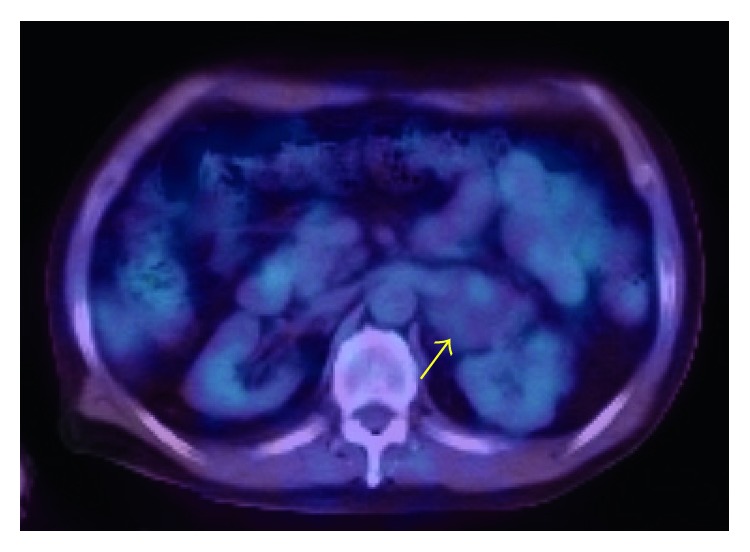
PET-CT revealed no uptake at the tumor (arrow).

**Figure 3:**
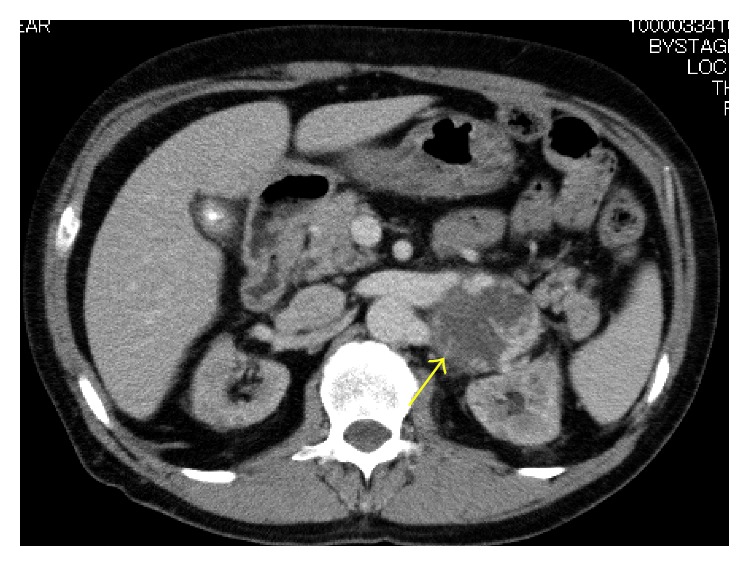
Contrast-enhanced CT. Contrast CT revealed slightly enhancement around the tumor (arrow).

**Figure 4:**
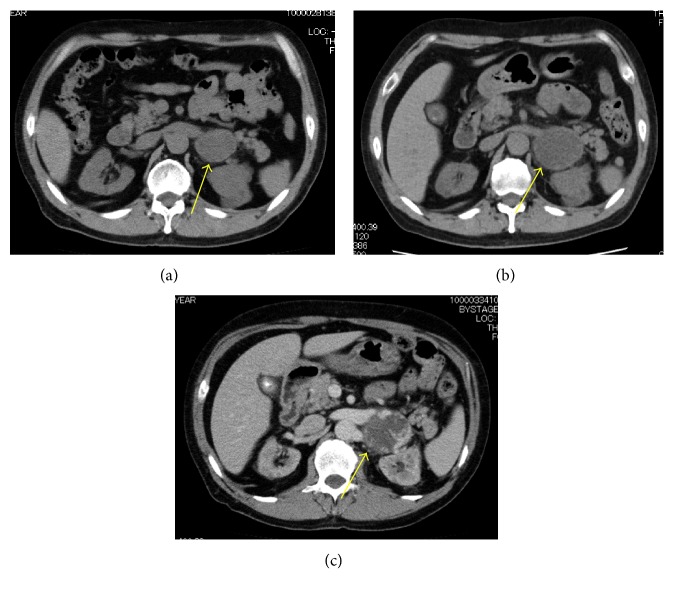
CT images. The tumor developed gradually.

**Figure 5:**
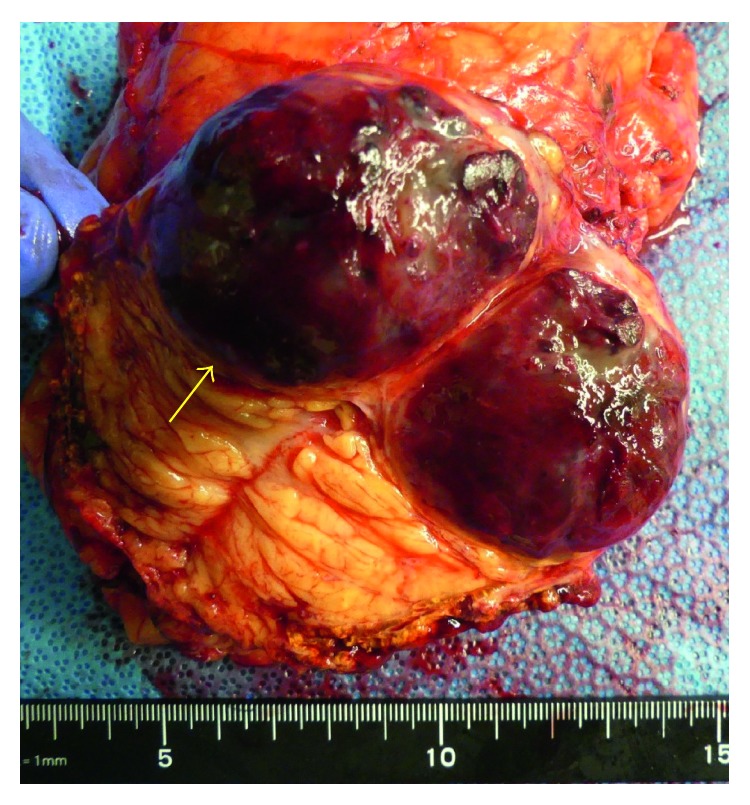
Resected tumor was capsuled (arrow).

**Figure 6:**
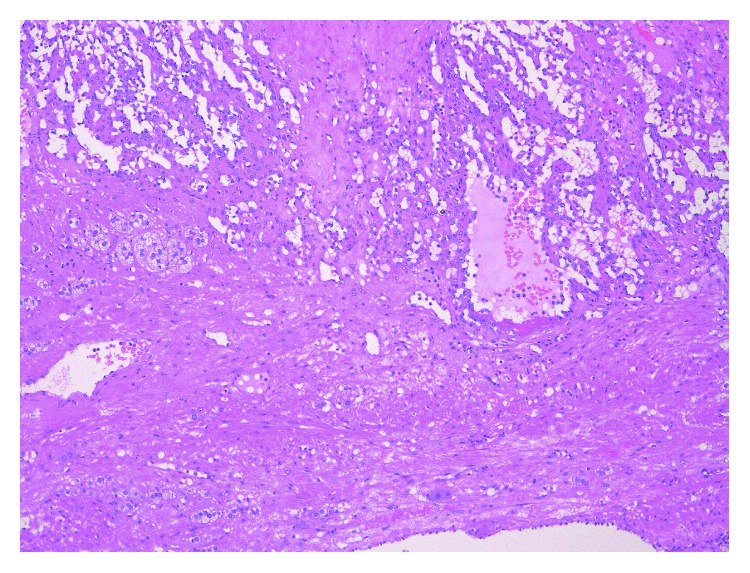
Hematoxylin and eosin staining.

CT showed a retroperitoneal mass of 4.3 cm in diameter in April 2016. Because of the patient's low renal function, enhanced CT was not performed. Gd-enhanced magnetic resonance imaging showed low intensity on T1-weighted imaging (T1WI) and high intensity on T2WI (Figure 1). Positron-emission tomography (PET)-CT showed no uptake in the mass (Figure 2). These imaging findings indicated no evidence of malignancy, so routine follow-up was planned. The tumor grew to nearly 6.0 cm in November 2016. After starting hemodialysis, enhanced CT suggested hemangioma due to the high degree of enhancement in the tumor at an early phase with an irregular cystic lesion (Figure 3). Preoperative imaging findings, a hormonal examination, and the tumor marker levels suggested hemangioma. Due to the tumor's growth and induction of hemodialysis, we decided to perform tumor resection (Figure 4). If the patient had not already started hemodialysis, adrenalectomy might have been an option (Figures 5 and 6).

The patient underwent retroperitoneal tumor and renal resection and was discharged seven days postoperatively. The resected tumor was solid and dark blown in color, measuring 50 × 37 × 30 mm in size (Figure 5). Histologically, it consisted of small vessels covered with monostromatic endothelium, which was positive for CD34 immunohistochemically. Some vessels were rather large and dilated. No atypia or mitosis was noted in endothelial cells (Figure 6). Adrenal cortices were seen around the tumor, so the final diagnosis was adrenal hemangioma. The patient was free from recurrence seven months postoperatively.

## 3. Discussion

Typical symptoms of adrenal hemangioma include pain, nausea, and vomiting, but 70%–80% of patients show no symptoms due to a lack of specific phenomena [1, 4]. Recently, most reported cases of adrenal hemangioma have been diagnosed incidentally based on imaging findings. On ultrasonography, cystic fluid usually shows as low echoic lesions, while hemorrhaging of cysts sometimes shows as high echoic lesions. Dynamic enhanced CT reveals increased enhancement in the area surrounding the tumor (early edge enhancement) and delayed central enhancement. MRI shows decreased intensity on T1WI and increased intensity on T2WI. CT and MRI show an iris phenomenon [5–9]. Based on these findings, a preoperative diagnosis is difficult to make.

Treatments for adrenal hemangioma in the previously reported cases included conservative observation and tumor resection. In cases with typical findings by CT or MRI, conservative therapy was performed. However, when imaging findings suggested adrenal hemangioma, surgical treatment was selected for larger tumors because of the difficulty of a preoperative diagnosis. Grumbach and Yamakita reported the indications of surgical resection. Grumbach et al. suggested surgical resection be performed for adrenal tumors >6 cm in diameter, given the 25% incidence of malignant diseases; no apparent indications have been established for tumors 4 to 6 cm in size [10]. Yamakita et al. reported that the incidence of malignancy was 3.8% for tumors <3 cm in size and 6.6% for those 3 to 6 cm in size; resection is suggested for tumors >3 cm in size [11].

In our case, although the tumor size was 43 mm, careful observation was selected due to suspicion of adrenal hemangioma based on the CT and MRI findings.

Nakagawa and Fuzimoto reported that, in cases of a large tumor size, adrenal hemangioma accompanied malignant tumor (tumor size: 8.5 and 24.0 cm, resp.) [12, 13]. Therefore, even in cases suspected of being adrenal hemangioma, resection should be performed for large tumors.

## 4. Conclusion

We herein report a case of adrenal hemangioma that was difficult to diagnose preoperatively. Due to the enlargement of the tumor during follow-up, the patient had to undergo tumorectomy.

## Data Availability

Due to ethical restrictions, the raw data underlying this paper are available upon request from the corresponding author.
